# *Acinetobacter*—the bad, the ugly, but also the good!

**DOI:** 10.1128/msphere.00010-26

**Published:** 2026-02-06

**Authors:** Santiago Castillo-Ramírez, Rafael López-Sánchez, Humberto Peralta

**Affiliations:** 1 Programa de Genómica Evolutiva, Centro de Ciencias Genómicas, Universidad Nacional Autónoma de México 61740 , Cuernavaca, México; Universita degli Studi di Napoli Federico II, Naples, Italy

**Keywords:** *Acinetobacter*, genomics, metagenome-assembled genomes (MAGs), *Acinetobacter calcoaceticus*

## Abstract

The genus *Acinetobacter* is vast and diverse regarding its hosts. However, it is best known as an opportunistic pathogen that causes hard-to-treat nosocomial infections. Yet, some species of the genus can be beneficial for some hosts. Such is the case of *Acinetobacter calcoaceticus*, which can have a significant impact on tomato plants, as was recently shown in a paper by Robertson et al. (S. Robertson, A. Mosca, S. Ashraf, A. Corral, et al., mSphere 11:e00842-25, 2026, https://doi.org/10.1128/msphere.00842-25). Importantly, that study also exemplifies how metagenomics in general, but metagenome-assembled genomes in particular, can be employed to understand the functional specialization and identity of the bacterial species dwelling in particular environments.

## COMMENTARY

*Acinetobacter* is a bacterial genus with huge metabolic diversity (1), which can be found in many different sources, including animals, humans, plants, soil, and diverse aquatic environments (2, 3). However, the genus is mostly recognized as an opportunistic pathogen and source of hard-to-treat nosocomial infections (4, 5)—the bad and the ugly. Nonetheless, even the most nefarious species of the genus, *Acinetobacter baumannii*, has a habitat that extends well beyond causing illness in humans and can be found in many hosts or sources (6). Importantly, although not very much known or publicized, some species of the genus can be beneficial for some organisms—the good. Such is the case of *Acinetobacter calcoaceticus*, which is considered a plant growth-promoting bacterium (7–11). In this regard, a recent paper published in this journal shows that *A. calcoaceticus* can impact the growth of an important staple crop, namely the tomato (12).

The authors skillfully use a battery of metagenomics approaches to characterize the microbiota of tomato plants. Using 16S rRNA amplicon sequencing, the authors show a microhabitat partition considering the phyllosphere, rhizosphere, and root interior. Notably, at the plant-soil continuum, an *Acinetobacter* sp. comes out as a principal component of the microbiota. Then, to understand this phenomenon, they conduct shotgun metagenomics of the rhizosphere and noted a group of rhizosphere-enriched functions, including adaptability to salinity and mobilization of mineral nutrients. Finally, they carry out a metagenome-assembled genomes (MAGs) analysis and pinpoint *A. calcoaceticus* as the relevant species, but also identify the genes implicated in the functional specialization. Among this functional specialization, the authors found phosphate solubilization, reactive oxygen species detoxification, and siderophore production.

Without a doubt, genome sequencing has been very important to understand the biology and dispersion of many pathogenic and some non-pathogenic *Acinetobacter* species (2, 13). For instance, *Acinetobacter junii* was recently described as a One Health emerging pathogen in a global genomic epidemiology study (14); another example is the genomic characterization and evolution of the understudied nosocomial pathogen *Acinetobacter pittii* (15). Yet, going beyond whole-genome sequencing (WGS), this mSphere article (12) clearly shows the full potential of metagenomics to characterize the ecology and biology of understudied *Acinetobacter* species. It is worth remembering that metagenomics circumvents the culture dependence necessary for many microbial characterization assays, and typical WGS is dependent on bacterial culture. An aspect to highlight from this recently published *mSphere* article is the use of MAGs to finely identify the actual bacterial species implicated in the phenomenon under study (12). In this regard, very recently, MAGs have become a very promising tool to expand our knowledge of the habitat of some *Acinetobacter* species (16–18). Along these lines, this work and other recent studies (16–18) clearly show the potential of using MAGs to analyze populations of *Acinetobacter* from unconventional sources that may not be amenable to bacterial culture. For instance, a recent study found *A. baumannii* MAGs from rather non-typical sources, such as activated sludge or the New York City subway (18). The possibility of studying atypical sources, hard to study with conventional bacterial culture approaches, is of paramount importance if we are to truly understand the different habitats the *Acinetobacter* species can dwell in, or for that matter, any other bacterial species . We anticipate that in the coming years, as the construction of MAGs improves and becomes more standardized, the use of this approach is bound to be an important tool to better characterize the habitat and functional versatility of many *Acinetobacter* species.
